# Meta-Analysis of Randomized Controlled Trials of the Effects of Tai Chi on Blood Pressure

**DOI:** 10.1155/2020/8503047

**Published:** 2020-10-07

**Authors:** Xiaosheng Dong, Meng Ding, Xiangren Yi

**Affiliations:** ^1^Department of Sport and Health, School of Physical Education, Shandong University, Jinan 250061, China; ^2^College of Physical Education, Shandong Normal University, Jinan 250014, China

## Abstract

**Objectives:**

The purpose of this study was to investigate the influences of Tai Chi on blood pressure (BP) using the meta-analysis.

**Methods:**

This paper used 6 e-resource databases, and randomized controlled trials on the role of Tai Chi on blood pressure were retrieved. Besides, the meta-analysis was conducted according to the guidelines of the Moose-recommendations and applied with Review Manager 5.3, and the risk of bias assessment was performed with the Cochrane Collaboration's tool. The inclusion, data extraction, and risk of bias assessment were independently finished by two researchers.

**Results:**

There are 24 trials meeting the criteria of inclusion and the results were reviewed. The meta-analysis indicates that, compared with no exercise, Tai Chi had the influence of lowering systolic blood pressure (mean difference = −6.07, 95%CI (−8.75, −3.39), *P* < 0.00001) and diastolic blood pressure (mean difference MD = −3.83, 95%CI (−4.97, −2.69), *P* < 0.00001). No significant discrepancies in all outcomes between Tai Chi and other aerobic exercises were discovered.

**Conclusion:**

Tai Chi can significantly reduce systolic and diastolic pressure than inactivity. However, Tai Chi does not show advantages in reducing blood pressure compared to other aerobic exercises. The trial is registered with CRD42020175306.

## 1. Background

Hypertension is a chronic noninfectious disease with the highest economic burden in the world [1]. In epidemiological studies, hypertension is considered to be an essential risk factor for cardiovascular mortality and morbidity [2, 3], and its complications are now the most common cause of death in western countries [4]. Despite the significant progress in monitoring and drug treatment of hypertension [5], inadequate blood pressure control is still a serious public health problem at home and abroad [6].

Modern medical studies show that physical activity can decrease blood pressure and stop the occurrence of hypertension [7, 8], and physical activities are connected with a variety of health benefits, including a reduction in the incidence of cardiovascular disease and stroke [9, 10]. Studies have shown that physically active people have a lower blood pressure than sedentary people [11] and an organized work-out plan is recommended for people with elevated blood pressure or essential hypertension [12]. To sum up, exercise therapy has become an effective means of control and therapy of blood pressure.

Tai Chi is originated in ancient China. In order to promote health and longevity, it is a traditional fitness method [13]. It has elements such as body movement, idea guidance, and breath control and can achieve the function of body and mind adjustment [14]. These exercises may be more effective in maintaining neuroendocrine balance and controlling blood pressure. Few published studies systematically evaluated the effect of Tai Chi on blood pressure, believing that Tai Chi could reduce blood pressure to a certain extent [15–17].

However, there are still some inadequacies in these studies. One is that the involved studies included RCT and prospective studies, and the sources of evidence were inconsistent. Second, although systematic evaluation was made, the meta-analysis was not conducted. Third, the research is relatively early, and some high-quality research literature is not included. In addition, there is no evidence that Tai Chi, as a type of physical and mental adjustment, has an advantage over other aerobic sports in reducing blood pressure. Therefore, it is necessary to include more high-quality studies with systematic evaluation and meta-analysis to further clarify the influence of Tai Chi on blood pressure.

## 2. Methods

The protocol for this review was registered in the International Prospective Register of Systematic Reviews, PROS-PERO (registration number: CRD42020175306).

### 2.1. Search Strategies

This paper used the electronic resource database to search MEDLINE via PubMed, The Cochrane Library, Web of Science, CNKI, Wan Fang Data, and VIP to retrieve randomized controlled experiments on the effect of the intervention of Tai Chi on blood pressure regulation. The search covered all data up to January 2019, and a bibliography of the articles was included to prevent omissions. The keywords searched were separated into a target (blood pressure) search and intervention (Tai Chi) search, which were adjusted in the specific database. All searches combined topic search with free search. Search strategies were based on multiple studies. Specific search terms included Taiji, Tai Chi, Taijiquan, blood pressure, hypertension, BP, systolic blood pressure, and diastolic blood pressure.

### 2.2. Inclusion and Exclusion Criteria

Inclusion criteria are as follows: English and Chinese studies of randomized controlled trials were included; subjects were not limited to age or gender; systolic and diastolic blood pressure were tested; Tai Chi was taken as the main intervention measure in the experimental group; the control group did no exercise or other aerobic exercises (such as jogging or walking except Tai Chi) or resistance exercises; the subjects had no serious hypertension-related complications.

Exclusion criteria are as follows: these nonrandomized controlled trials were grouped on a voluntary basis; studies did not include indicators (systolic blood pressure, diastolic blood pressure) to be discussed in this study; Tai Chi or control groups contained antihypertensive drugs or nutrients or other nonconventional therapies, including herbal medicine, acupuncture, moxibustion, cupping, and massage; the main exercise in the experimental group was not Tai Chi; or, subjects had serious hypertension-related complications.

### 2.3. Trial Inclusion and Data Extraction

Trials inclusion and data extraction were independently completed by the two researchers. In the process of paper inclusion and data extraction, a third researcher was asked to analyze and decide whether to include and extract the data if any disagreements occurred. The following information was extracted: publication time, published journals, first author, trial names, intervention measures of experimental and control group, duration of intervention, number of control and experimental groups, particulars (age, gender) of subjects, and systolic and diastolic blood pressure indicators.

### 2.4. Risk of Bias Assessment

In the process of extracting data, the Cochrane Collaboration's tool was used to assess the bias risk of the included research. The evidence was based on studies ranked as having an either low, unclear, or high risk of bias. The risk of bias evaluation was independently performed by two researchers. The third rater was involved in the case of disagreement.

### 2.5. Statistical Analysis

The meta-analysis was conducted according to the guidelines of the Moose-recommendations [18]. Review Manager 5.3 software provided by Cochrane was applied for meta-analysis. The heterogeneity test between the results of the included studies was *χ*2. Statistical homogeneity between the results of each study indicated that when *P* > 0.01 and *I*^2^ < 50%, the fixed-effect model was adopted; when heterogeneity showed *P* < 0.01 and *I*^2^ > 50%, heterogeneity was analyzed by subgroup analysis. If there was no heterogeneity between subgroups, a fixed-effect model was used. Provided that statistical heterogeneity existed among subgroups in the included studies, a random effect model was used for meta-analysis. If there was too much heterogeneity between groups, descriptive analysis was conducted. Besides, a sensitivity analysis was applied if necessary.

## 3. Results

### 3.1. Trial Search

**Figure 1:**
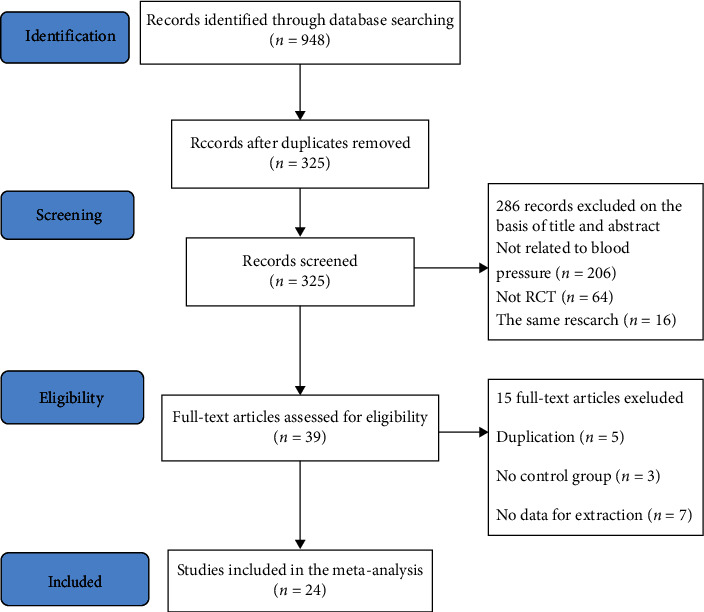
Flow diagram of study selection and identification.

In this paper, a randomized controlled trial of the effect of Tai Chi intervention on blood pressure was retrieved based on the criteria of exclusion and inclusion. 948 articles were found and were likely to be relevant, and 325 articles were retained after the repetitive articles were ruled out. Subsequently, 286 articles were deleted by reading the title and abstract, and 15 articles were excluded after the full text was reviewed against the eligibility criteria. Eventually, a total of 24 studies [19–42], including 6 English trials [19–24] and 18 Chinese trials [25–42], were selected. The selection process is shown in Figure 1.

### 3.2. Description of Studies

A total of 24 randomized controlled trials, with a total of 1608 subjects, were included. The average age of subjects ranged from 20.12 to 69.30 years. The minimum duration of follow-up was 8 weeks while the maximum duration of follow-up was 6 months. All patients in intervention groups mainly received Tai Chi as an intervention measure. 1 article adopted te Chen's Tai Chi and Yang's Tai Chi [37], 1 article adopted the Low Strength Tai Chi and Medium-intensity Tai Chi [39], and 22 articles did not reflect the classification of Tai Chi [19–36, 38, 40–42]. All included articles have reported frequencies and times of Tai Chi exercise. The control groups were separated into nonexercise groups and other aerobic exercise groups. The exercise of other aerobic exercise groups was walking and/or Yoga [22, 24, 25, 28, 29, 31, 32, 38, 41] (Table 1).

### 3.3. Risk of Bias Assessment

**Figure 2:**
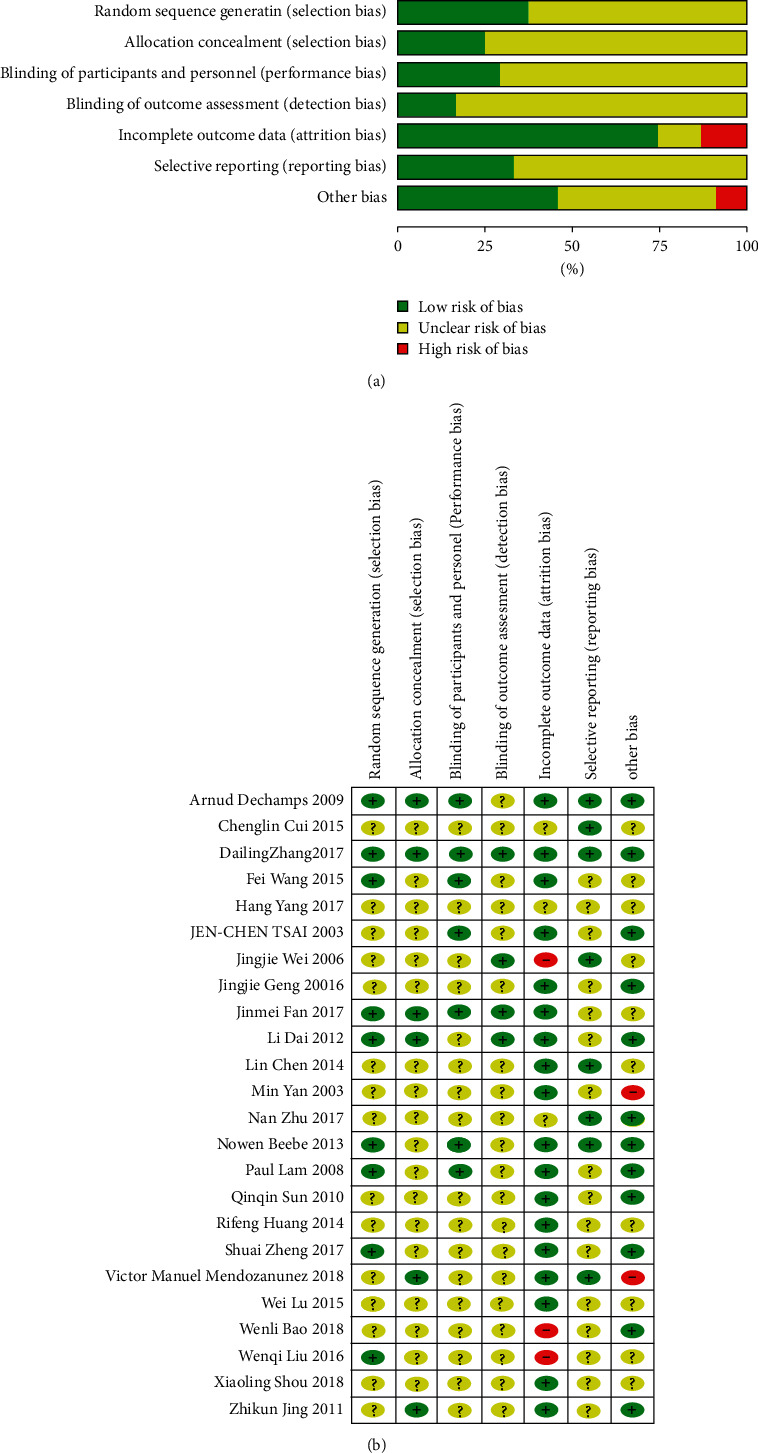
Assessment of risk of bias: (a) risk of bias graph and (b) risk of bias summary.

The results of the Cochrane Collaboration's tool are that with regard to random sequence generation, 9 studies were rated low risk, and another 15 were rated unclear risk. With regard to allocation concealment, 4 studies were rated low risk, and another 20 were rated unclear risk. With the blinding of participants and personnel, 5 studies were rated low risk, and another 19 were rated unclear risk. With regard to blinding of outcome assessment, 3 studies were rated low risk, and another 21 were rated unclear risk. With regard to incomplete outcome data, 15 studies were rated low risk, 6 were rated unclear risk, and another 3 were rated high risk. With regard to selective reporting, 1 study was rated low risk, and another 23 were rated unclear risk. With regard to other biases, 2 studies were rated low risk, 20 were rated unclear risk, and the other 2 studies were rated low risk (Figure 2).

### 3.4. Outcomes

#### 3.4.1. Systolic Blood Pressure

**Figure 3:**
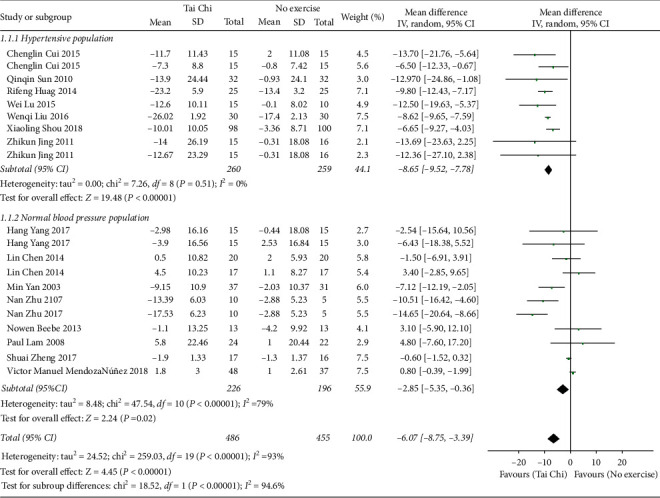
The meta-analysis for comparing SBP between Tai Chi and no exercises.

*(1) Tai Chi versus No Exercise*. At first, the data of 16 related studies (*n* = 1017) [19–21, 23, 24, 26, 27, 30, 33–37, 39, 40, 42] could be pooled, but the data heterogeneity was quite severe. After the analysis of subgroups according to the blood pressure status, it was found that the heterogeneity in the normal blood pressure population was as high as 96%. According to the sensitivity analysis of the data, it was inferred that the data of Jen-Chen Tsai 2003 was the principal source of heterogeneity in this subgroup. Therefore, the data in this study was subsequently excluded, and the other data studies were pooled. Finally, a data combination was conducted with the random effect model, which revealed that, compared with nonexercises, Tai Chi showed a better function in lowering SBP, and the difference was statistically significant [MD = −6.07, 95% CI (−8.75, −3.39), *P* < 0.00001]. The subgroup analysis showed that the hypertensive population and normal blood pressure population had statistical significance (Figure 3).

**Figure 4:**
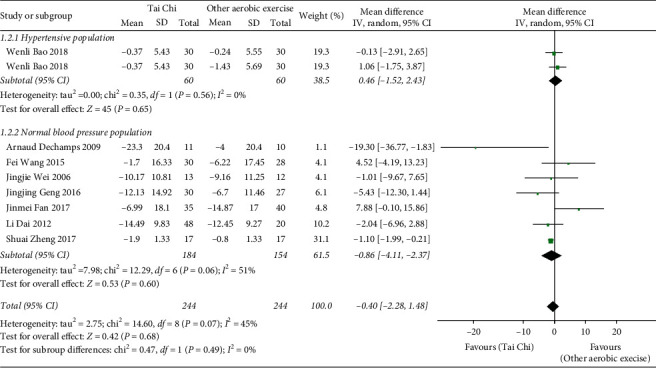
The meta-analysis for comparing SBP between Tai Chi and other aerobic exercises.

*(2) Tai Chi versus Other Aerobic Exercises*. As exhibited in Figure 4, 9 relevant studies (*n* = 591) [22, 24, 25, 28, 29, 31, 32, 38, 41] allowed for data combined, but the data heterogeneity was relatively high. After the analysis of subgroups according to the blood pressure status, it was found that the heterogeneity in the hypertension population was as high as 94%. By means of sensitivity analysis of the data, it was inferred that the data of Dailiang Zhang 2017 and Wenli Bao 2018 (fitness walking) were the major source of heterogeneity in the subgroup. Therefore, the data in this study were subsequently excluded, and the other data studies were pooled. Eventually, the data of 8 studies (*n* = 458) was combined, and the results demonstrate that, in comparison with other aerobic exercises, the difference was of no statistical significance [MD = −0.40, 95% CI (−2.28, 1.48), *P*=0.68].

#### 3.4.2. Diastolic Blood Pressure

**Figure 5:**
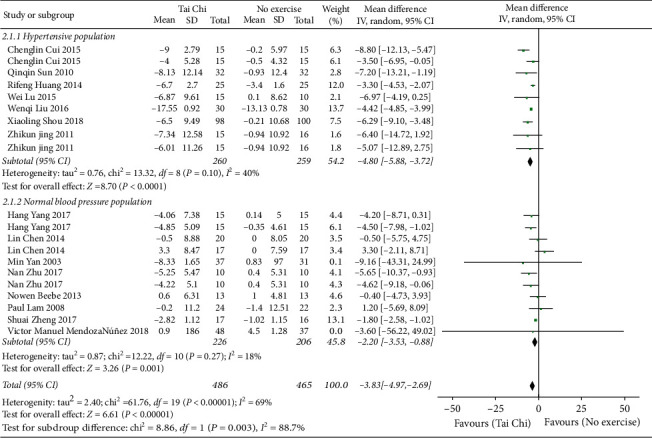
The meta-analysis for comparing DBP between Tai Chi and no exercises.

*(1) Tai Chi versus No Exercise*. Initially, the data of 16 studies (*n* = 1017) [19–21, 23, 24, 26, 27, 30, 33–37, 39, 40, 42] was pooled, but the data heterogeneity was too high. Through the analysis of subgroups based on the blood pressure status, it was found that the heterogeneity in the normal blood pressure population was as high as 71%. According to the analysis of the sensitivity of the data, it was deduced that the data of Jen-Chen Tsai 2003 was the major source of heterogeneity in the subgroup. Thus, this study's data was excluded. Finally, the data of 15 studies (*n* = 941) was pooled, and it showed that compared with nonexercises, Tai Chi showed a greater role in reducing SBP, and the difference was of statistical significance [MD = −3.83, 95% CI (−4.97, −2.69), *P* < 0.00001]. The subgroup analysis showed that the hypertensive population and normal blood pressure population had statistical significance (Figure 5).

**Figure 6:**
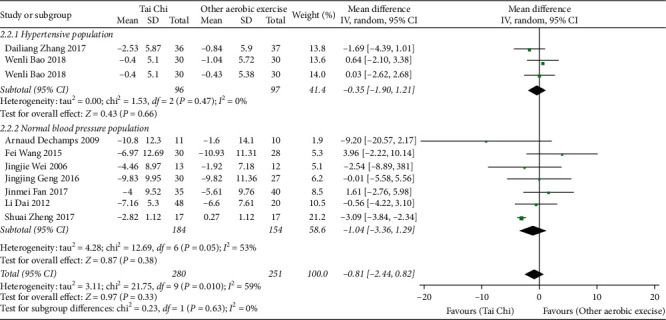
The meta-analysis for comparing DBP between Tai Chi and other aerobic exercises.

*(2) Tai Chi versus Other Aerobic Exercises*. It is shown in Figure 6 that 9 related studies (*n* = 591) [22, 24, 25, 28, 29, 31, 32, 38, 41] allowed for data combined, but the data heterogeneity was very high. Through the analysis of subgroups according to the blood pressure status, it was found that the heterogeneity in the hypertension population was as high as 80%. According to the analysis of the sensitivity of the data, it was deduced that the data of Wenli Bao 2018 (fitness walking) were the principal source of heterogeneity in the subgroup. The data of this study were finally excluded accordingly, and the data of other studies were pooled. Finally, the data of 9 studies (*n* = 531) were pooled, and compared with other aerobic exercises, the difference was of no statistical significance (MD = −0.81, 95% CI (−2.44, 0.82), *P*=0.33).

### 3.5. Publication Bias Evaluation between Tai Chi and No Exercise

In our research, the diastolic blood pressure for Tai Chi and the nonexercise indicator was analyzed through funnel plots. A total of 15 trials, with 951 patients, were incorporated, which showed that it was asymmetric for funnel plot of included studies distribution, demonstrating that potential publication bias exists when Tai Chi was compared with nonexercise.

### 3.6. Publication Bias Evaluation between Tai Chi and Other Aerobic Exercises

Funnel plots were applied to analyze the systolic blood pressure, which contains data from 8 studies and 458 patients. The results reflected that it was asymmetric for funnel plot of included studies' distribution, demonstrating that potential publication bias exists when Tai Chi was compared with no exercise.

## 4. Discussion

This study discussed the influence of Tai Chi on blood pressure through systematic evaluation and meta-analysis. According to the results of this study, Tai Chi has a better effect on reducing systolic and diastolic blood pressure than no exercise. These results are basically consistent with those of Yeh et al. [15–17]. It indicates that Tai Chi, as a traditional exercise, can be used to control blood pressure and assist in the treatment of hypertension.

According to the relevant literature, traditional exercises such as qigong, Tai Chi, and yoga all have certain effects on the management of blood pressure levels [43–46]. Tai Chi is a kind of traditional Chinese exercise, which is characterized by looseness, steadiness, slowness, and evenness and is a moderate or low-intensity aerobic exercise [47]. In this study, Tai Chi showed a significant advantage in both systolic and diastolic blood pressure compared with no exercise, and according to the subgroup analysis of blood pressure status, we found that Tai Chi had a significant effect on lowering blood pressure in both hypertensive and healthy blood pressure groups. Therefore, Tai Chi has a lowering effect on blood pressure and can be used for primary and secondary prevention of hypertension [48]. Although the results of this study showed that Tai Chi showed no advantage in reducing systolic or diastolic blood pressure compared with other aerobic exercises, previous studies have confirmed the effect of aerobic exercise on lowering blood pressure [49]. Therefore, Tai Chi can also be used to control blood pressure and assist in the treatment of hypertension [50]. It is worth noting that people with prehypertension or high blood pressure may find that it is difficult to stick to a regular exercise program, whereas Tai Chi is a gentle and easy exercise to perform. In addition, studies have shown that Tai Chi's perseverance is better than other exercises [51, 52]. Besides, Tai Chi is easy to learn and moderate in intensity, requires less space, and can be practiced alone or in groups. These characteristics are not available in many other aerobic exercises. Therefore, Tai Chi may be a safe and effective alternative to traditional exercise therapy.

At present, the prevention and treatment of hypertension are the management of habits, mainly including diet and exercise. As far as the exercise intervention for patients with hypertension is concerned, it is recommended that patients perform moderate-intensity aerobic training with heart rate maintained at 65–75% of the maximum heart rate for 3–5 days a week, 30 minutes a day, or 90–150 minutes a week [53]. Besides, individualized exercise programs should be offered based on the patient's willingness and ability. It is worth noting that the general mechanical and simple exercise can make patients boring, directly affecting the implementation of the exercise program, while Tai Chi, as part of the traditional Chinese medicine, mainly emphasizes the combination of breathing and body movement. It can be used as an auxiliary means in the prevention and treatment of high blood pressure.

Our study found that Tai Chi had a significant benefit in lowering blood pressure compared to inactivity. This shows that Tai Chi has the effect of lowering blood pressure. Although this study suggests that Tai Chi has no obvious advantages than other aerobic exercises, relevant studies showed that subjects have good adherence to Tai Chi, and such perseverance is better than the standard movement considering that patients with prehypertension or hypertension are very difficult to maintain a regular exercise program [15]. Therefore, Tai Chi is an effective exercise replacement therapy in lowering blood pressure.

Limitations of this study shall also be taken into account. First, limited by objective conditions, only MEDLINE via PubMed, The Cochrane Library, Web of Science, CNKI, Wan Fang Data, and VIP were applied, so potential omissions may occur; second, the quality of included papers was low, and the random distribution method and blind method were not described; third, due to the large heterogeneity of the included studies, only random effect model can be used, which will have a certain impact on the results; fourth, the frequency and intensity of Tai Chi and other aerobic exercises also differ greatly among the studies, which may produce some heterogeneity and affect the analysis results; fifth, the included studies have a large possibility of publication bias.

## 5. Conclusion

Tai Chi can significantly reduce systolic and diastolic pressure than inactivity. Therefore, Tai Chi can be applied in the process of blood pressure control, prevention, and adjuvant treatment of hypertension. However, Tai Chi does not show advantages in reducing blood pressure compared to other aerobic exercises.

## Figures and Tables

**Table 1:** Characteristics and quality assessments of the included trials.

First author, year	Participants (total, E/C)	Age (mean (SD), E/C)	Hypertension	Experimental intervention	Control	Duration, sessions with supervision per week, time per session
Min Yan 2003	68, 37/31	20.12 (1.08)/20.72 (0.64)	No	Tai Chi	N	6 months, 3–5, 40–8-min
Jen-Chen Tsai 2003	76, 37/39	51.6 (16.3)/50.5 (9.8)	No	Tai Chi	N	12 weeks, 3, 50 min
Jingjie Wei 2006	25, 13/12	21.62 (1.45)/21.58 (1.44)	No	Tai Chi	FW	8 weeks, 5, 30 min
Paul Lam 2008	46, 24/22	63.2 (8.6)/60.7 (12.2)	No	Tai Chi	N	1–3 months, 2, 60 min 4–6 months, 1, 60 min
Arnaud Dechamps 2009	21, 11/10	44.4 (11.9)	No	Tai Chi	AE	10 weeks, 1, 120 min
Qinqin Sun 2010	64, 32/32	57.19 (8.09)/57.25/5.63	Yes	Tai Chi	N	3 months, 6, 90 min
Zhikun Jing 2011	31, 15/16	58.47 (9.65)/59.12 (6.26)	Yes	Low Strength Tai Chi	N	10 weeks, 3, 60 min
Zhikun Jing 2011	31, 15/16	57.53 (6.35)/59.12 (6.26)	Yes	Medium-intensity Tai Chi	N	10 weeks, 3, 60 min
Li Dai 2012	68, 48,20	68.10 (5.27)/69.30 (5.91)	No	Tai Chi	Walking	12 weeks, 5, 30 min
Nowen Beebe 2013	26, 13/13	60.4 (6.2)/62.6 (5.9)	No	Tai Chi	N	16 weeks, 3, 45 min
Rifeng Huang 2014	50, 25/25	62.5 (7.5)	Yes	Tai Chi	N	8 weeks, 7, 45 min
Lin Chen 2014	34, 17/17	53.2 (9.8)/57.3 (11.2)	No	Tai Chi	N	3 months, 3, 90 min
Lin Chen 2014	40, 20/20	53.35 (9.69)/58.45 (11.50)	No	Tai Chi	N	3 months, 3, 90 min
Fei Wang 2015	58, 30/28	37.47 (8.41)/41.57 (10.53)	No	Tai Chi	BG	3 months, 5, 45 min
Wei Lu 2015	25, 15/10	62.00 (3.59)	Yes	Tai Chi	N	16 weeks, 6, 60 min
Chenglin Cui 2015	30, 15/15	60.0 (7.5)/61.4 (3.5)	Yes	Tai Chi	N	3 months, 3, 60 min
Chenglin Cui 2015	30, 15/15	62.9 (6.0)/63.2 (7.6)	Yes	Tai Chi	N	3 months, 3, 60 min
Jingjing Geng 2016	57, 30/27	34 (7)/38 (5)	No	Tai Chi	AE	3 months, 5, 45 min
Wenqi Liu 2016	60, 30/30	56.33 (7.16)/56.80 (6.78)	Yes	Tai Chi	N	12 weeks, 5, 40 min
Shuai Zheng 2017	33, 17/16	35.4 (2.1)/34.6 (2.3)	No	Tai Chi	N	12 weeks, 1, 120 min
Shuai Zheng 2017	34, 17/17	35.4 (2.1)/32 (1.8)	No	Tai Chi	AE	12 weeks, 1, 120 min
Dailiang Zhang 2017	73, 36/37	66.33 (4.74)/67.51 (4.09)	Yes	Tai Chi	Walking	12 weeks, 3, 60 min
Hang Yang 2017	60, 30/30	45.9 (0.7)	No	Tai Chi	N	16 weeks, 3, 90 min
Jinmei Fan 2017	75, 35/40	62.80 (8.10)/60.45 (8.13)	No	Tai Chi	AE	12 weeks, 5, 60 min
Nan Zhu 2017	20, 10/10	59.76 (5.21)/60.11 (5.59)	No	Chen's Tai Chi	N	3 months, 5, 60 min
Nan Zhu 2017	20, 10/10	60.78 (5.56)/60.11 (5.59)	No	Yang's Tai Chi	N	3 months, 5, 60 min
Víctor Manuel MendozaNúñez 2018	85, 48/37	67.4(4.7)/68.2 (6.6)	No	Tai Chi	N	6 months, 5, 50 min
Xiaoling Shou 2018	198, 98/100	52.35 (3.26)/51.35 (4.21)	Yes	Tai Chi	N	3 months, 7–14, 45–60 min
Wenli Bao 2018	60, 30,30	47.83 (5.35)/46.98 (5.15)	Yes	Tai Chi	AE	20 weeks, 3-4, 60 min
Wenli Bao 2018	60, 30/30	47.83 (5.35)/47.12 (4.90)	Yes	Tai Chi	Yoga	20 weeks, 3-4, 60 min
Wenli Bao 2018	60, 30/30	47.83 (5.35)/47.22 (5.15)	Yes	Tai Chi	Walking	20 weeks, 3-4, 60 min

E: experimental group; C: control group; N: no exercise; NR: not reported; min: minute; SD: standard deviation; FW: fitness walking; BG: Broadcast Gymnastics; AE: aerobic exercise.

## Data Availability

Specific study data are available from the authors on request.

## Abbreviations

BP: Blood pressure
SBP: Systolic blood pressure
DBP: Diastolic blood pressure
CI: Confidence interval
SD: Standard deviation
RCT: Randomized controlled trial.
